# Standardizing Virtual Healthcare Deployment: Insights From the Implementation of Telerobotic Ultrasound to Bridge Healthcare Inequities in Rural and Remote Communities Across Canada

**DOI:** 10.1177/21501319251329314

**Published:** 2025-03-27

**Authors:** Amal Khan, Sandro Galea, Ivar Mendez

**Affiliations:** 1 Department of Community Health & Epidemiology, University of Saskatchewan, Saskatoon, SK, Canada; 2 Virtual Health Hub, Saskatoon, SK, Canada; 3 Washington University in St. Louis, St. Louis, MO, USA; 4 Department of Surgery, University of Saskatchewan, SK, Canada

**Keywords:** virtual healthcare, health inequities, rural and remote communities, Indigenous health, digital literacy, community profiles, readiness assessments, telerobotic ultrasonography, healthcare access

## Abstract

The COVID-19 pandemic has accelerated the integration of virtual care into healthcare systems, presenting a unique opportunity to address healthcare inequities in rural and remote communities, particularly those that are Indigenous. This commentary outlines critical steps and best practices for deploying virtual care in underserved regions, drawing on over a decade of experience in Saskatchewan. Key recommendations include creating detailed community profiles, assessing digital literacy, and using standardized readiness tools to evaluate infrastructure and clinical needs. A weighted prioritization framework ensures efficient resource allocation, while partnerships with Indigenous-led institutions, such as SIIT, equip local healthcare assistants to support virtual care delivery. Examples from successful telerobotic ultrasonography deployments in the rural and remote communities of Saskatchewan highlight the potential of virtual care to improve healthcare access, outcomes, and sustainability. By tailoring interventions to community-specific contexts and involving local stakeholders, *virtual care* can bridge health disparities and serve as a replicable model for similar settings worldwide.

## The Role of Virtual Care in Redesigning Rural Healthcare

The advent of the COVID-19 pandemic has catalyzed the integration of virtual care into in-person care, creating an opportunity to redesign the healthcare system to be more efficient, increase capacity, reduce costs, and improve both patient and provider experiences. This transformation is especially relevant in rural and remote communities, many of which are Indigenous.^1^ These communities have long faced social exclusion, health disparities, and significant healthcare inequalities, which have contributed to worsened health outcomes.^1,2^ Virtual care could offer a viable pathway to improve healthcare outcomes and reduce health disparities. For virtual care to be effective, a standardized approach must be used to deploy it in underserved communities.

## Deploying Virtual Care: Lessons From Saskatchewan

We deployed a variety of virtual healthcare technologies, including telerobotic ultrasound, to improve healthcare delivery in rural and remote communities. This commentary highlights critical steps and best practices for implementing virtual care in rural and remote settings, drawing from experiences in Saskatchewan that can serve as a model for similar initiatives elsewhere.^3-8^ While the outlined steps provide a generic framework for introducing virtual care technologies in underserved areas, we capitalize on the deployment of telerobotic ultrasound as a detailed example to support the discussion that follows.

## Understanding Community Needs for Virtual Care Implementation

The deployment of virtual care must begin with a deep understanding of the social patterning of health, which includes social exclusion, health disparities, and inequalities that rural and remote populations face. A key foundational step in the effective deployment of virtual care is identifying the unique needs and characteristics of each rural community. This process is particularly critical for Indigenous communities that are diverse in their historical and cultural contexts. Developing community profiles is essential, involving collecting comprehensive data such as demographic information, prevalent health conditions, socioeconomic status, cultural frameworks, digital literacy, and existing healthcare and communications infrastructure. These profiles serve as a vital resource, enabling healthcare providers and policymakers to tailor virtual care solutions that address the specific health priorities, barriers, opportunities, and resource gaps of each community. Digital literacy has been considered a new determinant of health. Understanding the digital literacy levels within a community is key to guiding the selection of appropriate technological platforms and the design of user-friendly interfaces that ensure equitable widespread adoption and effective use of virtual care services.^9^

## Assessing Readiness and Overcoming Infrastructure Barriers

Equally important is the utilization of screening tools like checklists and readiness assessments. These can be instrumental in identifying healthcare priorities and evaluating the preparedness of communities for virtual care interventions. We have recently developed a comprehensive evaluation tool^10^ that provides a structured approach to assessing community capacity and readiness. This tool considers clinical needs, technology infrastructure, and human resources/workflows, providing actionable insights that enhance local engagement and successful implementation. For example, in many rural and remote areas, the lack of adequate communications infrastructure has posed significant challenges to the success of virtual care initiatives.^11^ A standardized screening process that evaluates technological and telecommunications readiness and infrastructural capacity can help overcome these obstacles, ensuring that virtual care is effectively implemented and addresses the most pressing healthcare needs. In areas with limited internet connectivity, exploring alternative technological solutions, such as satellite-based communication systems, can ensure that virtual care remains accessible to all community members.

## Strategic Prioritization of Virtual Care Interventions

Once the community’s needs and technological capabilities are clearly understood, establishing a criterion-based system for prioritizing interventions is necessary. This involves developing a weighted framework that considers the severity of healthcare needs, existing infrastructure, technological readiness, and the potential impact on the community. Such an analysis ensures that resources are allocated efficiently and that healthcare priorities are addressed to maximize community benefits. We have used this approach to successfully deploy telerobotic ultrasonography services to 6 remote communities in Saskatchewan.^5-8,12^ This initiative was guided by a thorough assessment of community needs and technological readiness, allowing for the successful implementation of advanced robotic diagnostic imaging technologies that significantly improved access to specialized care in these underserved areas.

## Capacity Building Through Workforce Training

Following this strategic prioritization, the next phase involves the deployment of virtual care services, requiring careful planning, such as the provision of training for healthcare providers and community members while establishing clear clinical pathways and protocols for virtual consultations. We have partnered with Saskatchewan postsecondary institutions that train healthcare workers, such as the Saskatchewan Indian Institute of Technologies (SIIT), to develop a Virtual Care Assistant applied certificate program.^13^ Indigenous learners are at the core of SIIT, representing over 90% of the student body. Graduates of this program will be equipped with the necessary skills to support clinicians in remotely delivering virtual healthcare services and locally assisting patients in accessing virtual care in their home communities. This approach not only addresses healthcare needs but also creates opportunities for Indigenous community members to actively participate in the delivery of virtual care.

## Ensuring Community Engagement and Cultural Sensitivity

The implementation of virtual care must be deeply rooted in the community, with local stakeholders actively involved to ensure that the programs are culturally sensitive and tailored to meet the specific healthcare needs of the population. Engaging these stakeholders enhances the cultural relevance of the programs and fosters a sense of ownership and commitment among community members, which is essential for the long-term sustainability of virtual care services.

## Evaluating Virtual Care Outcomes Across Multiple Domains

To evaluate the effectiveness of virtual care programs, a comprehensive set of outcome measures should be applied, covering clinical, economic, and environmental dimensions. Clinically, metrics such as patient and clinician satisfaction, health outcomes, and access to care should be tracked to assess the impact of virtual care on the overall health and well-being of the community. For example, a reduction in missed appointments and an increase in follow-up visits are indicators of improved access to care, which is particularly significant in rural and remote areas where healthcare services are often scarce. Economically, the cost-effectiveness of virtual care compared to traditional models should be analyzed, considering factors such as reduced travel costs for patients and the potential for more efficient use of healthcare resources.^8^ Environmentally, the reduction in the carbon footprint due to decreased travel for in-person visits contributes to the broader goal of sustainable healthcare delivery.^14^ These outcome measures provide a comprehensive understanding of the impact of virtual care and help identify areas for improvement, ensuring that programs continue to evolve and meet the changing needs of the community.^15^

## Virtual Care Benefits in Underserved Communities

Evidence from virtual care initiatives demonstrates measurable improvements in healthcare access, cost-effectiveness, and provider efficiency, particularly in underserved communities. The deployment of telerobotic ultrasonography in Saskatchewan’s remote areas has significantly reduced the need for patient travel, with studies showing that approximately 70% of telerobotic ultrasound examinations provided sufficient diagnostic quality, minimizing referrals to distant healthcare facilities and reducing wait times for essential imaging services.^12^ From an economic standpoint, telehealth interventions have proven to be cost saving, particularly by eliminating unnecessary travel and optimizing specialist consultations. Research indicates that telehealth can reduce costs when system-funded patient travel is prevented, as well as when early intervention through virtual care prevents more complex and costly procedures.^16^ Additionally, virtual consultations have been linked to improved patient adherence to care, with studies showing a 13% reduction in missed appointments, enhancing both patient outcomes and healthcare providers’ efficiency.^17^ These findings highlight the tangible benefits of virtual care, reinforcing its role as a sustainable and effective solution for addressing healthcare disparities in rural and remote regions.

## Discussion

### Overcoming Barriers to Virtual Care Adoption

The successful adoption of virtual care in rural and remote communities depends on addressing several key challenges that could hinder its effectiveness. *Healthcare provider resistance* is a notable concern, often driven by uncertainties regarding workflow integration, increased administrative responsibilities, and doubts about the clinical reliability of virtual consultations. Overcoming this requires structured provider training, well-defined clinical protocols, and strong evidence demonstrating the benefits of virtual care in improving patient outcomes. *Technological limitations*, such as unreliable internet access, power outages, and equipment malfunctions, pose additional risks, particularly in remote areas where digital infrastructure is often inadequate. Enhancing connectivity through broadband expansion, incorporating backup solutions like satellite-based communication, and ensuring technical support can help mitigate these disruptions. *Data security and patient privacy* are also critical considerations, as virtual healthcare platforms handle sensitive medical information. Ensuring compliance with privacy regulations, strengthening encryption measures, and upholding Indigenous data sovereignty principles will be essential for building trust and maintaining confidentiality. By proactively addressing these challenges, virtual care can become a reliable and sustainable solution for enhancing healthcare access in underserved communities.

### Comparative Insights from Other Regions

Virtual care deployment in Saskatchewan offers a valuable case study, but lessons from other regions provide additional insights into scalability and adaptability. Countries such as Australia and Norway have pioneered virtual healthcare models in remote regions, addressing challenges similar to those in Canada’s northern and rural communities. Australia’s Royal Flying Doctor Service^18^ integrates telehealth with mobile clinical outreach, ensuring continuity of care in isolated areas, while Norway^19^ has leveraged strong digital infrastructure to enhance virtual consultations in sparsely populated regions. Unlike these models, Saskatchewan’s approach focuses on telerobotic ultrasound integration and structured readiness assessments to prioritize interventions based on community-specific needs. However, shared challenges persist across jurisdictions, including digital infrastructure gaps, provider resistance, and data security concerns. A standardized yet adaptable framework, informed by diverse regional experiences, is essential to optimizing virtual care implementation and ensuring equitable healthcare access in remote populations.

## Conclusion: Future Research and Global Adaptability

Deploying virtual care in rural and remote communities requires a systematic and standardized approach that addresses the unique needs and challenges of underserved populations. By developing detailed community profiles, applying readiness assessments, prioritizing interventions based on a weighted criteria system, and ensuring well-planned implementation with community involvement, virtual care can effectively bridge gaps in healthcare access and improve health outcomes. The experiences from initiatives such as the telerobotic ultrasonography services in rural and remote communities of Saskatchewan provide valuable insights into the importance of a structured and community-centric approach to virtual healthcare deployment. However, the successful application of these strategies in Saskatchewan does not guarantee seamless replication elsewhere. More research is needed to explore how these approaches can be adapted across different rural and remote contexts, accounting for variations in demographic, economic, and policy landscapes. By continuing to refine and expand on these best practices, virtual care has the potential to transform healthcare accessibility globally, ensuring that underserved communities, regardless of location, benefit from equitable and sustainable healthcare innovations.

## References

[1] Loppie C Wien F. Understanding Indigenous Health Inequalities through a Social Determinants Model. National Collaborating Centre for Indigenous Health; 2022.

[2] Markham R Hunt M Woollard R , et al. Addressing rural and Indigenous health inequities in Canada through socially accountable health partnerships. BMJ Open. 2021;11(11):e048053. 10.1136/bmjopen-2020-048053 PMC8609942 34810181

[3] Holt T Sari N Hansen G , et al. Remote presence robotic technology reduces need for pediatric interfacility transportation from an isolated northern community. Telemed J E Health. 2018;24(11):927-933. 29394155 10.1089/tmj.2017.0211

[bibr4-21501319251329314] Tanya H Gregory H Veronica M. Contemplating remote presence technology for culturally safe health care for rural indigenous children. AlterNative. 2019;15(1):31-33.

[bibr5-21501319251329314] Adams SJ Yao S Mondal P Lim H Mendez I Babyn P. Sociodemographic and geographic disparities in obstetrical ultrasound imaging utilization: a population-based study. Acad Radiol. 2022;29(5):650-662. 34452819 10.1016/j.acra.2021.07.012

[bibr6-21501319251329314] Adams SJ Babyn P Burbridge B Tang R Mendez I. Access to ultrasound imaging: a qualitative study in two northern, remote, Indigenous communities in Canada. Int J Circumpolar Health. 2021;80(1):1961392. 34347560 10.1080/22423982.2021.1961392 PMC8344228

[bibr7-21501319251329314] Adams SJ Burbridge B Chatterson L McKinney V Babyn P Mendez I. Telerobotic ultrasound to provide obstetrical ultrasound services remotely during the COVID-19 pandemic. J Telemed Telecare. 2022;28(8):568-576. 33076753 10.1177/1357633X20965422 PMC7576332

[8] Adams SJ Penz E Imeah B , et al. Economic evaluation of telerobotic ultrasound technology to remotely provide ultrasound services in rural and remote communities. J Ultrasound Med. 2023;42(1):109-123. 35906950 10.1002/jum.16070

[9] Arias López MDP Ong BA Borrat Frigola X , et al. Digital literacy as a new determinant of health: a scoping review. PLOS Digit Health. 2023;2(10):e0000279. 10.1371/journal.pdig.0000279 PMC10569540 37824584

[10] Deason JP Adams SJ Khan A Lovo S Mendez I. A comprehensive evaluation tool to assess community capacity and readiness for virtual care implementation. J Telemed Telecare. Published online November 19, 2024. doi:10.1177/1357633X241293854 PMC12569120 39563105

[11] Maciach M . Chapter 4: broadband connectivity in rural and remote communities: a persistent challenge heightened by COVID-19 and the move to remote learning for K–12 students. In: Brown B Roberts V Jacobsen M , et al., eds. Ethical Use of Technology in Digital Learning Environments: Graduate Student Perspectives, Volume 2. University of Calgary; 2021: 72–92.

[12] Adams SJ Burbridge B Chatterson L Babyn P Mendez I. A telerobotic ultrasound clinic model of ultrasound service delivery to improve access to imaging in rural and remote communities. J Am Coll Radiol. 2022;19(1 Pt B):162-171. 35033305 10.1016/j.jacr.2021.07.023

[13] Virtual Health Hub Assistant. SIIT. 2024. Accessed January 7, 2025. https://siit.ca/programs/virtual-health-hub-assistant/

[14] Patel KB Gonzalez BD Turner K , et al. Estimated carbon emissions savings with shifts from in-person visits to telemedicine for patients with cancer. JAMA Netw Open. 2023;6(1):e2253788. 10.1001/jamanetworkopen.2022.53788 PMC9890284 36719682

[15] Thiel CL Mehta N Sejo CS Qureshi L Moyer M Valentino V , et al. Telemedicine and the environment: life cycle environmental emissions from in-person and virtual clinic visits. NPJ Digit Med. 2023;6(1):87. 37160996 10.1038/s41746-023-00818-7 PMC10169113

[16] Snoswell CL Taylor ML Comans TA Smith AC Gray LC Caffery LJ. Determining if telehealth can reduce health system costs: scoping review. J Med Internet Res. 2020;22(10):e17298. 10.2196/17298 PMC7605980 33074157

[17] Adepoju OE Chae M Liaw W Angelocci T Millard P Matuk-Villazon O. Transition to telemedicine and its impact on missed appointments in community-based clinics. Ann Med. 2022;54(1):98-107. 34969330 10.1080/07853890.2021.2019826 PMC8725902

[18] The Royal Flying Doctor Service (RFDS). Flying Doctor telehealth. Accessed February 26, 2025. https://www.flyingdoctor.org.au/what-we-do/tele-health/

[19] Norwegian Ministry of Local Government and Modernisation. Digitalisation Strategy for the Public Sector 2019-2025. Norwegian Ministry of Local Government and Modernisation; 2019.

